# Individual Surgical Treatment of Stage IV Müller-Weiss Disease According to CT/MRI Examination: A Retrospective Study of 12 Cases

**DOI:** 10.3389/fsurg.2022.694597

**Published:** 2022-03-17

**Authors:** Wenzhou Liu, Yanbo Chen, Gang Zeng, Tao Yang, Mengjun Ma, Weidong Song

**Affiliations:** ^1^Department of Orthopedics, Sun Yat-sen Memorial Hospital, Sun Yat-sen University, Guangzhou, China; ^2^Department of Emergency, Sun Yat-sen Memorial Hospital, Sun Yat-sen University, Guangzhou, China; ^3^Department of Orthopedics, The Eight Affiliated Hospital, Sun Yat-sen University, Guangzhou, China

**Keywords:** Müller-Weiss disease, CT/MRI, clinical outcomes, stage IV, surgery

## Abstract

**Background:**

This study reported the individual surgical treatment of 12 cases with stage IV Müller-Weiss disease (MWD) according to CT/MRI examination.

**Methods:**

In total, 12 cases diagnosed with stage IV MWD in our hospital from 2015 to 2019 were included in the retrospective study. Relevant clinical outcomes were evaluated preoperatively and postoperatively.

**Results:**

The follow-up results showed satisfactory outcomes in all cases. All the cases were presented with tenderness and chronic pain on the midfoot dorsum, and three cases were also presented with tenderness and pain on the lateral side of the midfoot, in which calcaneal cuboid arthritis was revealed by CT/MRI. The American Orthopedic Foot and Ankle Society (AOFAS) scores elevated from 62.5 ± 6.8 (range: 53–74) preoperatively to 95.3 ± 7.2 (range: 73–100) postoperatively (*P* < 0.005). The Visual Analog Scale (VAS) scores declined from 4.2 ± 0.9 (range: 3–5.5) preoperatively to 0.5 ± 0.3 (range: 0–2) postoperatively (*P* < 0.001). On the weight-bearing lateral view of the foot, the Tomeno-Méary angle (TM lat) changed from −11.2 ± 4.2 (range: −17.2 to −2.8) degrees preoperatively to −2.4 ± 3.9 (range: −10.2 to 5.2) degrees postoperatively (*P* < 0.001).

**Conclusions:**

The fusion of the talus-navicular joint and the adjacent affected joint provide good clinical outcomes. The CT/MRI scans are helpful to identify the adjacent joint arthritis and provide indications for individual treatment for Stage IV MWD.

## Introduction

Müller-Weiss disease (MWD) is rare osteopathy in the foot navicular bone and is characterized by chronic pain on the dorsum of the hindfoot and/or midfoot, with weight-bearing deformation and lesion in the navicular bone, talonavicular arthritis, and progression into pes-planus with hindfoot *varus* deformity (1–3). The affected navicular is typically in comma shape and arthritis with varying degrees is presented at the naviculocuneiform and talonavicular joints (4, 5). The MWD diagnosis is based on clinical findings and weight-bearing plain radiographs of the foot. The main diagnostic method is weight-bearing radiographs of the foot and ankle. Maceira and Rochera presented a staging system by dividing the MWD into five stages according to the appearance of the navicular and the Meary-Tomeno (M-T) angle between the longitudinal axes of the first metatarsal and talus. According to the degree of deformity, the MWD can be classified into five stages: Stage I: the mild symptoms with normal radiographs and mild changes in navicular bone; Stage II: the dorsal intersection of the Tomeno-Méary (TM) angle with foot pain, and talar head and talipes cavus are laterally dislocated in radiography; Stage III: the split and compressed navicular bone and decreased medial longitudinal arch; Stage IV: the plantar intersection of the TM lines with the expulsion of a navicular bone fragment and hindfoot *varus* deformity, and the paradoxical *pes plano varus* is also presented; Stage V: the completely extruded navicular bone and formation of the talocuneiform joint.

Stage IV: the MWD is characterized by the increasing medial column collapse with arch height loss, hindfoot equinus paradoxical *pes plano varus*, M-T lines intersecting on the plantar side, and degeneration of the subtalar joint (typically the anterior facet) based on CT scan (6). In addition, the CT is useful in assessing deformity and arthritis, and MRI is useful in detecting bone marrow edema and effusion in adjacent joints. Thus, CT and MRI are important diagnostic strategies for MWD (7–9). In addition, CT and MRI are also useful for the differential diagnosis of oncological pathologies and secondary tumors, which, although rare, may involve the tarsal scaphoid (10). Surgical treatment is considered when the proper conservative management failed (11). The reported conservative treatment duration varies from 2 to 60 months (12). However, different studies reported that the results were poor before taking the surgical interventions (13–15). The surgical techniques include talonavicular-cuneiform (TNC) arthrodesis, talonavicular (TN) arthrodesis, triple arthrodesis, replacing destructed navicular bone with the autologous calcaneus bone, and calcaneal osteotomy (16–18). The TN and TNC joint fusion is the most popular surgical interventions (19, 20). In these studies, the disease was estimated mainly by X-ray, and treated with the same surgery method in one report. In the present study, we further reported 12 cases with stage IV MWD, which were treated with TN, TNC, and calcaneal-cuboid arthrodesis according to CT/MRI examination.

## Materials and Methods

### Patients

This study was approved by the Ethics Committee of our hospital. Informed consent was obtained from each patient. This study reviewed patients with staged IV MWD who received surgical treatment in our hospital from January 2015 to September 2019, retrospectively. The diagnosis of stage IV MWD was made based on radiological evaluation, medical history, and clinical examination. All the patients underwent anteroposterior (AP) and lateral weight-bearing radiograph of the foot and axis radiograph of the calcaneous preoperatively and postoperatively. The CT and/or MRI examinations were also applied in all patients preoperatively. Patients were admitted for surgeries with the following symptoms: complaint of midfoot pain for more than 6 months and failed conservative treatment for more than 2 months. All the patients diagnosed with MWD were reviewed in our database, and those diagnosed as stage IV were included in this study. Afterward, patients were excluded with the following criteria: (1) previous navicular traumatic fracture of navicular stress fracture; (2) rheumatoid arthritis; (3) infectious arthritis; and (4) Charcot arthropathy of the midfoot. One patient was excluded because of Charcot arthropathy of the midfoot (21).

### Surgical Procedures

The patient was placed in the supine position with a thigh tourniquet under spinal anesthesia. A cancellous autogenous bone graft was acquired from the ipsilateral iliac crest. A longitudinal dorsal incision was made lateral to the *extensor hallucis longus* tendon, then the *dorsalis pedis* artery and the deep peroneal nerve laterally, and the *extensor hallucis longus* tendon was retracted medially. The bony spurs, residual cartilage, and sclerotic bone from the involved bone were cut with an osteotome and cleared with a curette until healthy. A rough bone surface was prepared and several holes (2 mm in diameter) were drilled to facilitate the fusion, while the talus and the navicular bone were released and reduced as possible. The iliac cancellous bone graft was packed to fill the defect space between the talus and navicular bone, as well as the defect space of cuboid and calcaneal bone if necessary. Full thread hallow screws and locking plate were used for talus-navicular bone fixation, and full thread hallow screws were used for calcaneal-cuboid bone fixation. After the surgery, the foot was immobilized using plaster for 4 weeks, then, immobilized with a walking boot cast that allowed ankle movement for another 4 weeks, and a pain-tolerated non-full weight-bearing was allowed gradually in the following 4 weeks. A full weight-bearing was not allowed until 12 weeks after the surgery.

### Radiographic Evaluation and Follow-Up

**Figure 1:**
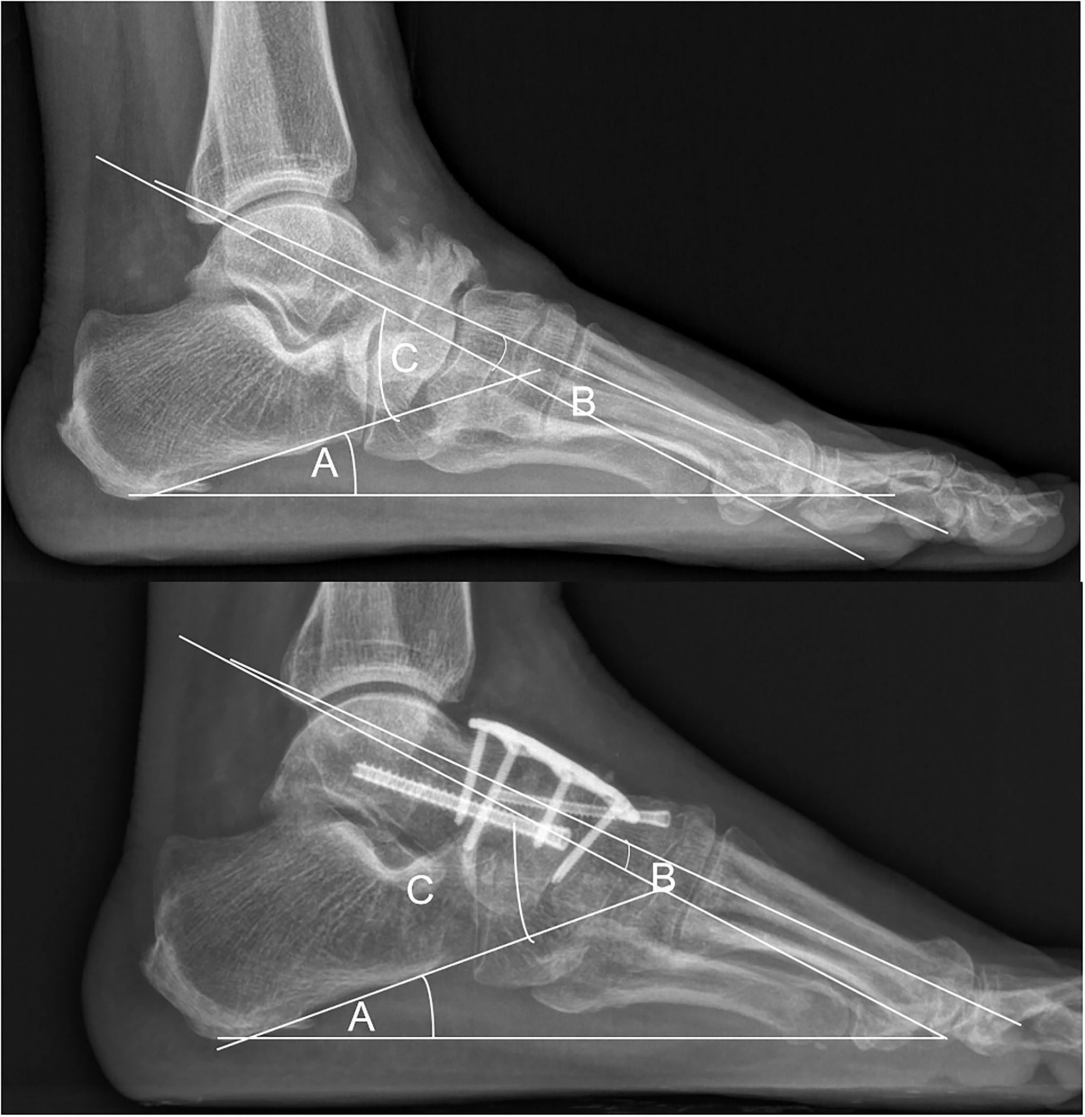
Weight-bearing lateral plain radiograph. (A) The calcaneal pitch angle (CPA). (B) The Temeno-Méary angle in this case was a dorsiflexion angle, given a negative value (TM lat, plantar flexion = positive value, dorsiflexion = negative value). (C) The lateral talocalcaneal angle (TAC lat).

**Figure 2:**
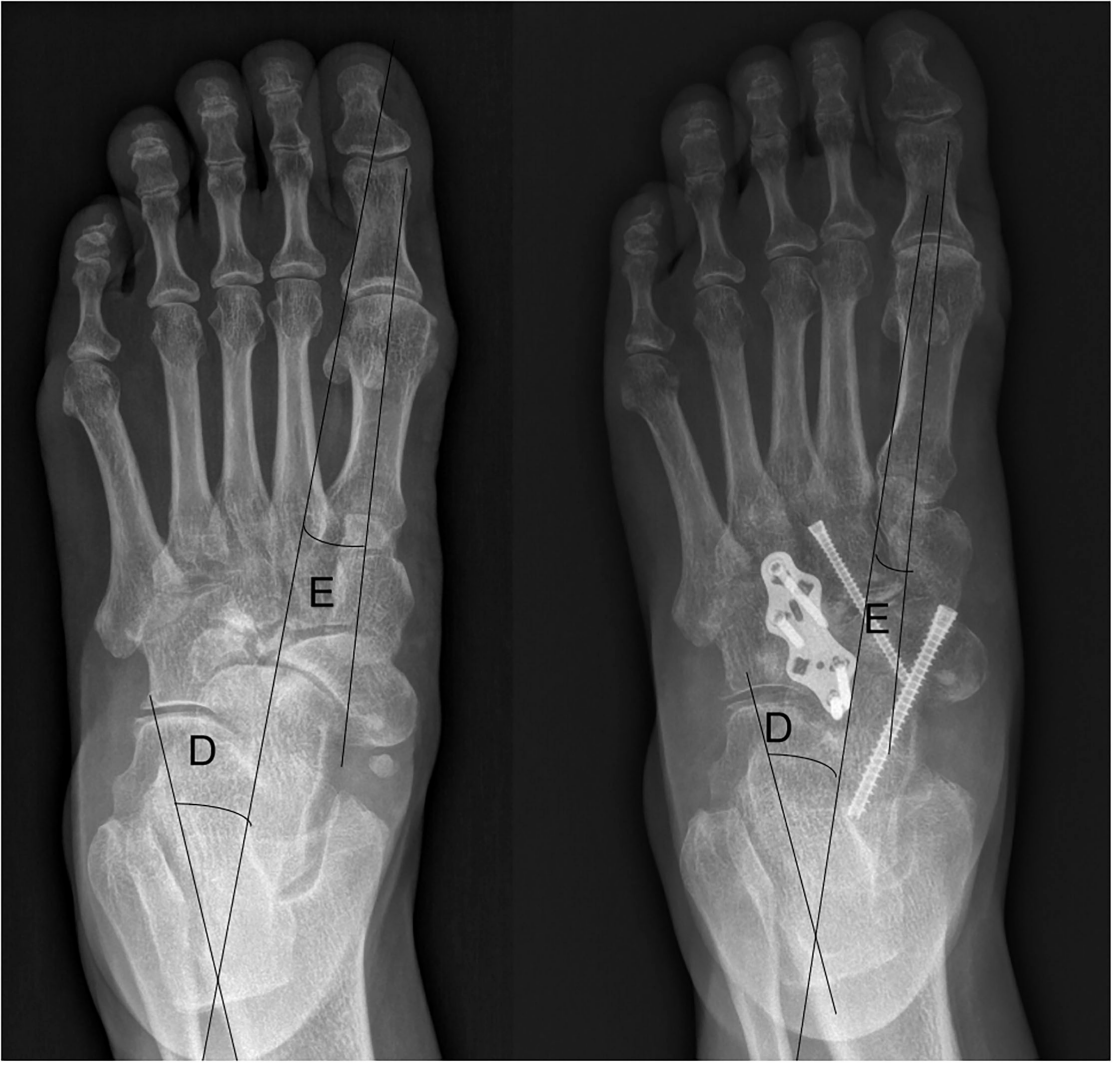
Weight-bearing anteroposterior plain radiograph in a case that underwent TNC arthrodesis. (D) The talocalcaneal angle (TCA ap). (E) The talar–first metatarsal angle (TM ap, abduction = positive value, adduction = negative value).

**Figure 3:**
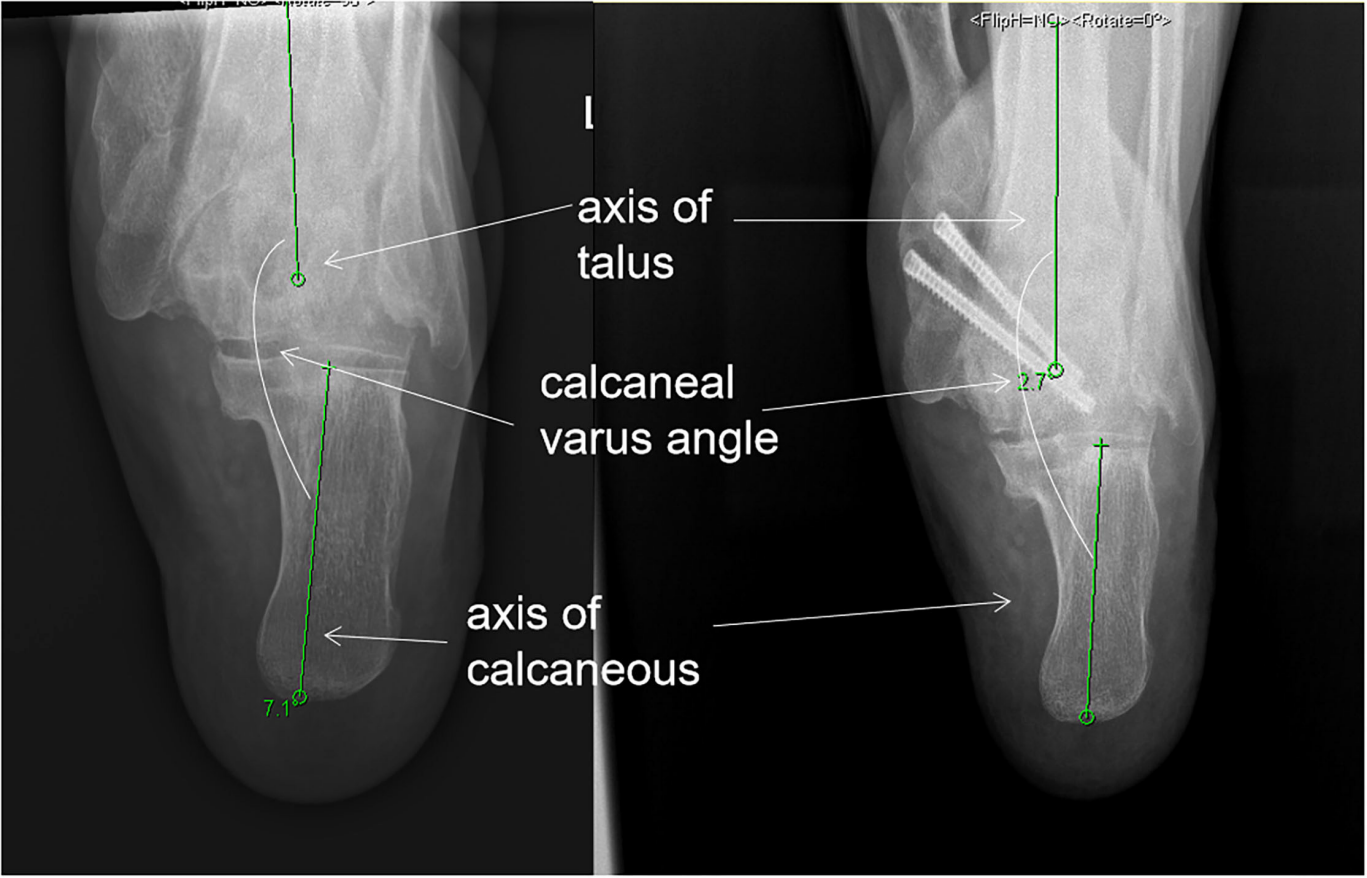
Calcaneal *varus* angle: the intersection angle between the calcaneus and tibia was measured preoperatively and postoperatively in the calcaneus axis radiography. We name the novel angle the “calcaneus *varus* angle” (CVA) (varus = positive value, valgus = negative value). The angle, in this case, was *varus*, thus, it was given a positive value.

The radiographic parameters on the weight-bearing anteroposterior plain radiograph [including the talar–first metatarsal angle (TM ap, abduction = positive value, adduction = negative value)], the talocalcaneal angle (TCA ap), and parameters on the weight-bearing lateral plain radiograph [including the calcaneal pitch angle (CPA) the lateral talocalcaneal angle (TAC lat)], and the Tomeno-Méary angle (TM lat, plantar flexion = positive value, dorsiflexion= negative value) were measured preoperatively and 3 months after the surgery. Figures 1, 2 illustrate the weight-bearing AP, and the lateral views of the foot are shown. The intersection angle between the calcaneus and tibia was measured preoperatively and postoperatively in the calcaneus axis radiography; we named the novel angle the “calcaneus *varus* angle” (CVA) (*varus* = positive value, valgus = negative value) (Figure 3). The fusion was considered successful when the bone trabeculae lines were across the fusion site. The patients were required follow-up in the outpatient department at 1.5, 3, 6, and 12 months after surgery. The final follow-up was undergone from September to October 2019. Before the surgery and at the final interview, the patients were evaluated by the ankle-hindfoot scale of the American Orthopedic Foot and Ankle Society (AOFAS) midfoot scale and the Visual Analog Scale (VAS).

### Statistical Analysis

All the original data were carefully checked and recorded. Statistical analysis was performed using SPSS 22.0 (IBM). Significant differences between different groups were determined using a Student's *t*-test. *P* < 0.05 was regarded as statistically significant.

## Results

**Figure 4:**
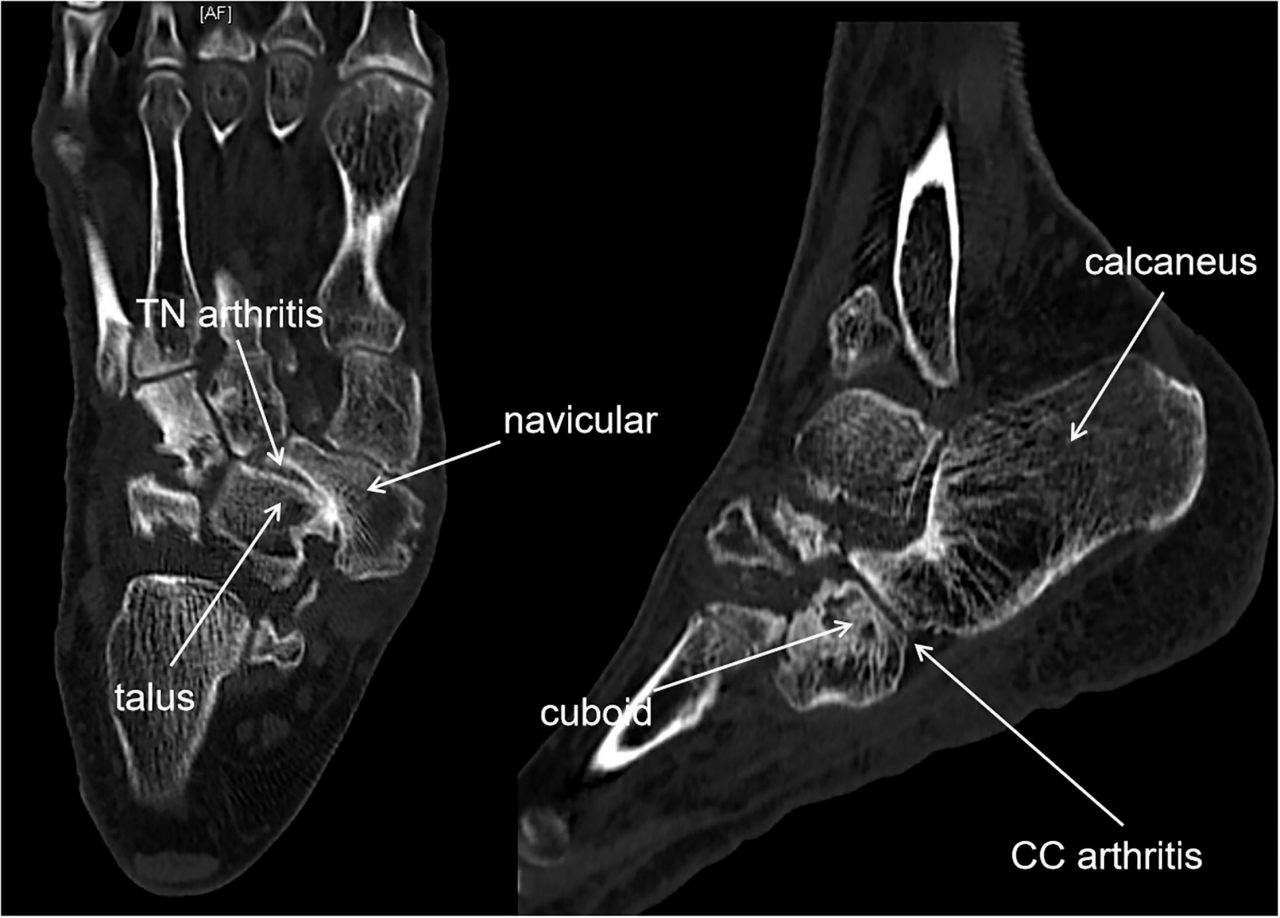
CT showed TN arthritis and calcaneal cuboid arthritis in a staged IV MWD, as well as navicular bone destruction and dislocation.

**Figure 5:**
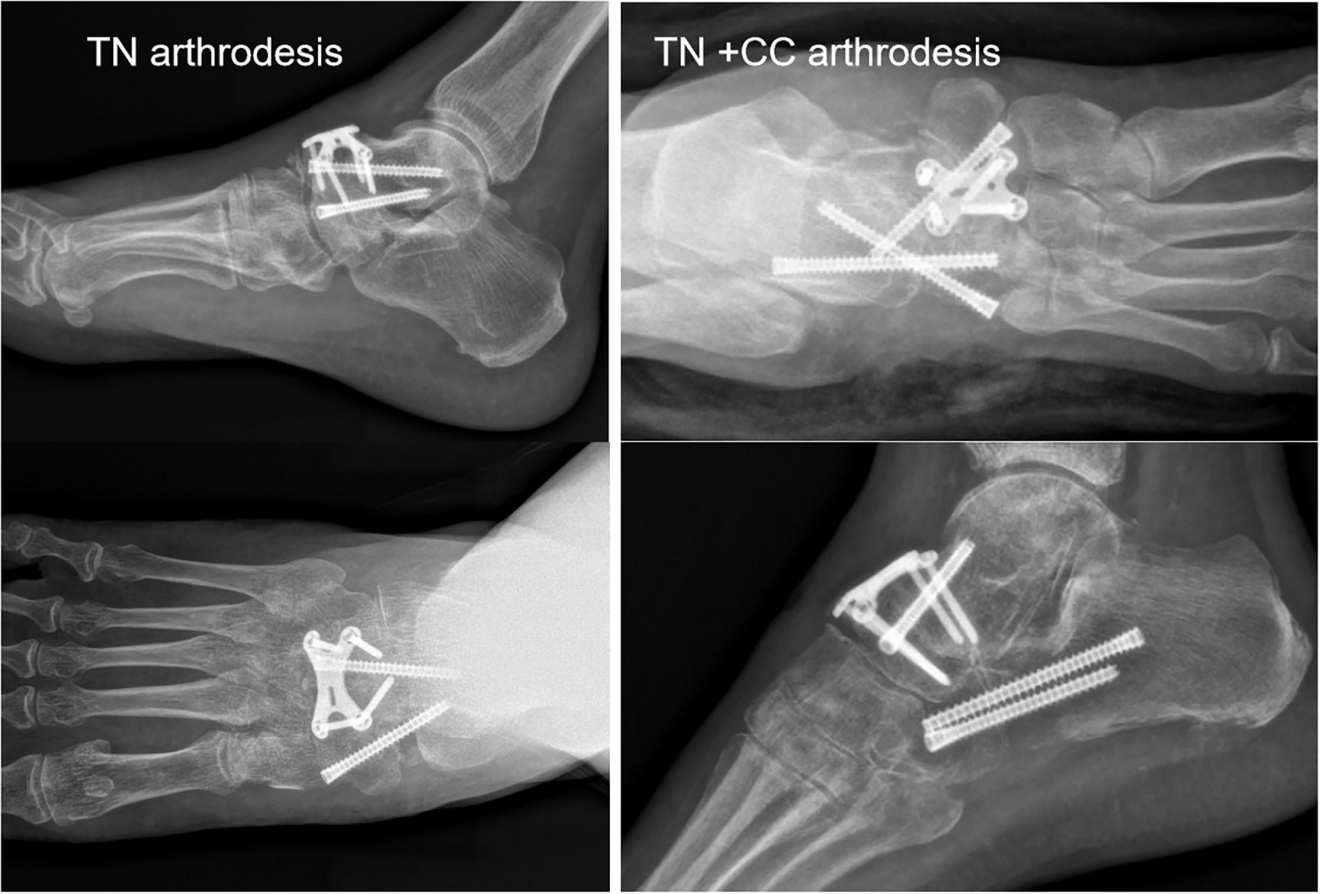
(Left) Anteroposterior (AP) and lat view of TN arthrodesis case. (Right) AP and lat view of TN + CC arthrodesis case.

**Figure 6:**
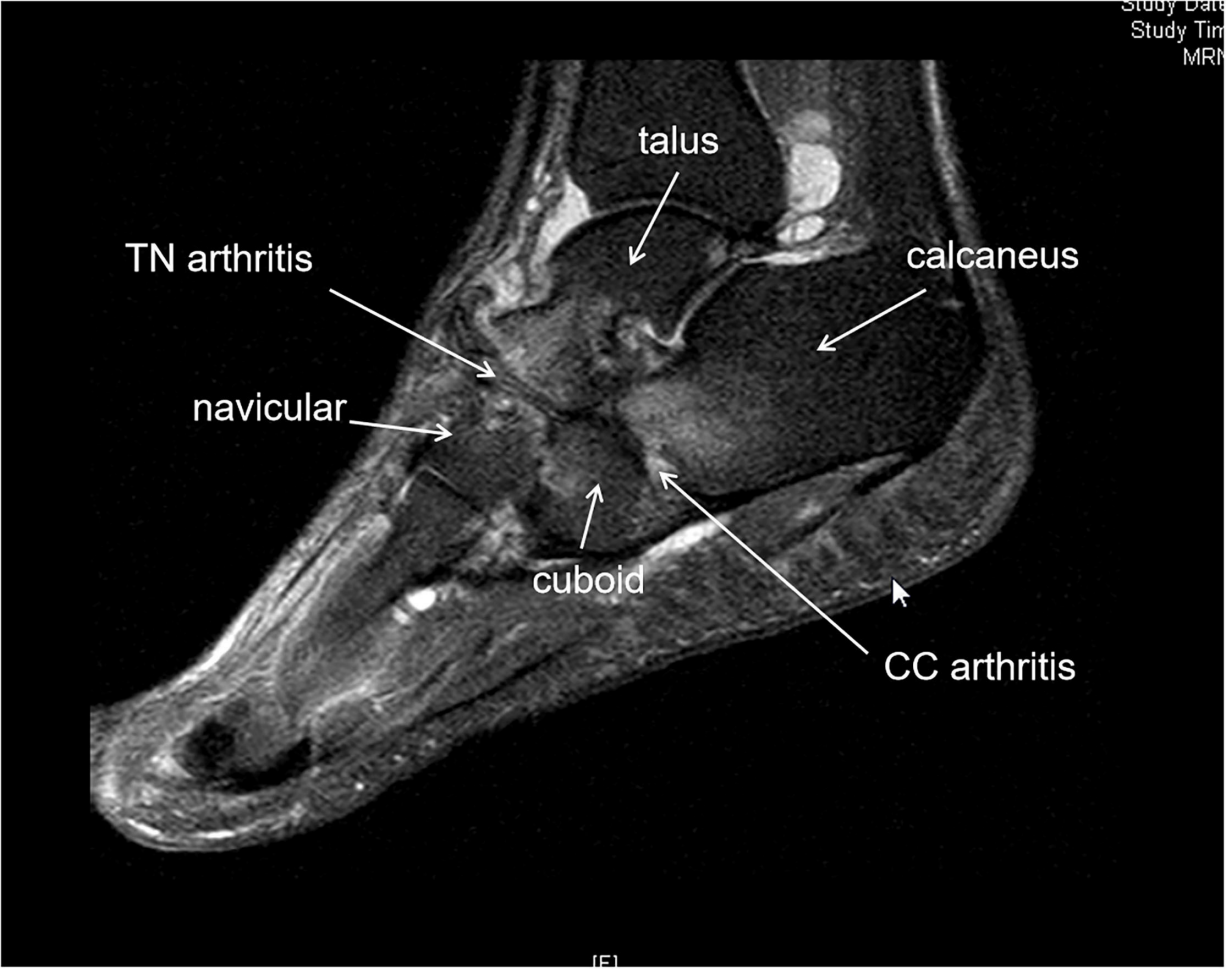
MRI showed talonavicular (TN) arthritis and calcaneal cuboid arthritis in a stage IV M-Weiss disease (MWD). Bone edema was observed in the navicular, talus head, calcaneous, and cuboid bone.

A total of 46 patients diagnosed with MWD were reviewed in our center, and 13 patients with stage IV MWD who received surgical treatment were identified. One of them was excluded due to Charcot arthropathy. Therefore, 12 cases (12 feet) were included for evaluation. Among these patients, all of them received MRI examination, and eight of them also received CT examination. MRI examination was more valid to identifying osteoarthritis than CT examination. Eleven of the twelve patients were female. The average age when the patients underwent operation was 58.3 ± 7.8 (range: 49–73) years old. Three feet were revealed to have calcaneal-cuboid arthritis by CT and/or MRI (Figures 4, 5) and underwent TN and calcaneal-cuboid (CC) arthrodesis, eight patients underwent TN fusion (Figure 5), and one underwent TNC fusion (Figure 6).

The follow-up duration ranged from 14 to 44 months with a mean of 27.5 ± 10.9 months. Table 1 illustrated the clinical features of the patients. The VAS and the AOFAS midfoot scale were determined before the surgery. All the patients were satisfied with the outcomes of the surgery at the last follow-up visit. No patient had an infection. One patient had skin necrosis after the surgery and the wound was healed after 2 months of the wound dressing. One patient was complicated with fixation failure at 2 months after the surgery, who admitted to full weight-bearing at 1.5 months post-surgery. She accepted revision surgery. She was required to take full weight-bearing until 4 months after the revision, and finally, had a satisfactory outcome. In the end, she achieved a bone fusion 8 months after the surgery. Two patients achieved bone fusion at 3 months post-surgery. The rest of the patients had bone fusion at 6 months post-surgery.

**Table 1:** General clinical characteristics of the patients.

**Case**	**Gender**	**Age (years)**	**Onset time (years)**	**Operation**	**Follow up (months)**	**Side**	**BMI**
1	F	62	2	TN fusion	42	R	23.3
2	F	60	1.5	TN fusion	37	L	26.2
3	F	49	3	TN fusion	44	L	24.9
4	F	69	0.5	TN fusion	41	R	25.2
5	F	68	10	TN + CC fusion	26	R	24.3
6	M	62	2	TN fusion	19	R	28.0
7	F	58	27	TN + CC fusion	14	R	27.7
8	F	50	3	TN + CC fusion	25	R	25.8
9	F	73	10	TN fusion	13	L	26.2
10	F	51	4	TNC fusion	26	L	21.6
11	F	50	4	TN fusion	17	L	31.0
12	F	55	12	TN fusion	36	R	24.8

At the last follow-up, the AOFAS scores elevated from 62.5 ± 6.8 (range: 53–74) preoperatively, to 95.3 ± 7.2 (range: 73–100) postoperatively (*P* < 0.005). The VAS scores reduced from 4.2 ± 0.9 preoperatively to 0.5 ± 0.3 points postoperatively (*P* < 0.001) (Table 1). At the end of the follow-up visit, 100% of union was achieved.

Nine of the 12 patients were presented with accessory navicular bone, however, no tenderness was found on the medial site of the navicular bone in all patients. The TM lat changed from −11.2 ± 4.2 (range: −17.2 to −2.8) preoperatively to −2.4 ± 3.9 (range: −10.2 to 5.2) degrees postoperatively (*P* < 0.001). The TAC lat decreased from 47.6 ± 7 (range: 36.8–56.5) preoperatively to 43.8 ± 7.1 (range: 29.4–59.3) degrees postoperatively (*P* = 0.14). The CPA increased from 7.8 ± 5.3 (range: 1.2–16.8) preoperatively to 10 ± 5.2 (range: 2.4–17.6) degrees postoperatively (*P* = 0.24). On the foot AP view, the TM ap decreased from 7.5 ± 4.4 (range: 1.1–16.6) preoperatively to 6.9 ± 3.9 (range: 0.6–14.4) degrees postoperatively (*P* = 0.35). The TCA AP changed from 17 ± 6.6 (range: 5.7–24.4) preoperatively to 16.9 ± 7.8 (range: 6.6–30.6) degrees postoperatively (*P* = 0.86). The angle between the calcaneus and tibia was preoperatively and postoperatively showed *varu*s angles in all patients, and the CVA decreased from 7 ± 4.7 (range: 1.0–15.2) preoperatively to 4.9 ± 4.8 (range: 0.5–14.5) degrees postoperatively (*P* = 0.21) (Tables 2, 3).

**Table 2:** Preoperative clinical and radiographic results.

**Case**	**AOFAS**	**VAS**	**TM lat (**°**)**	**TCA lat (**°**)**	**CPA (**°**)**	**TM ap (**°**)**	**TCA ap (**°**)**	**CVA (**°**)**
1	61	4	−13.60	42.70	1.20	7.10	5.70	15.00
2	53	3.5	−11.70	53.90	7.00	8.20	12.30	1.70
3	65	5	−11.30	56.50	14.20	16.60	6.20	9.20
4	65	4	−11.40	44.90	9.20	11.70	15.90	3.20
5	63	3	−11.30	56.70	10.60	10.40	23.40	15.20
6	74	5.5	−7.50	53.60	13.80	1.10	24.00	7.80
7	72	5	−17.20	36.80	7.80	5.80	24.30	1.00
8	50	5	−2.80	39.10	0.80	3.20	17.10	3.70
9	66	4	−17.00	48.10	2.70	10.80	24.40	5.10
10	60	3.5	−6.70	52.90	16.80	4.70	20.20	6.60
11	61	4.5	−8.80	40.30	2.90	8.40	13.10	5.20
12	60	5	−14.70	46.20	6.90	2.40	16.80	10.30

**Table 3:** Postoperative clinical and radiographic results.

**Case**	**AOFAS**	**VAS**	**TM lat (**°**)**	**TCA lat (**°**)**	**CPA (**°**)**	**TM ap (**°**)**	**TCA ap (**°**)**	**CVA (**°**)**
1	95	1	5.20	41.70	7.40	9.60	6.60	14.50
2	97	0	−2.80	45.00	8.70	3.40	7.30	0.50
3	97	0	−0.50	44.10	17.00	14.40	7.60	3.30
4	92	1	−2.70	46.20	8.80	11.50	14.80	1.90
5	97	1	−2.60	49.30	16.20	9.80	23.00	12.70
6	100	0	−1.40	58.30	15.20	2.40	25.20	7.70
7	100	0	−3.80	29.40	2.40	6.20	24.70	0.60
8	73	2	1.10	40.70	8.70	4.10	13.40	0.70
9	100	0	−10.20	43.10	3.50	9.10	30.60	1.20
10	95	1	−0.10	51.00	17.60	0.60	21.60	1.10
11	100	0	−2.90	36.70	4.10	7.10	11.40	5.90
12	97	0	−8.50	40.30	10.80	4.90	17.00	8.60

## Discussion

Surgical treatment is indicated for persistent symptoms after an adequate conservative treatment (2). Currently, there is no available consensus about the optimal surgical procedure for treating MWD. Arthrodesis is the most commonly used technique; while other techniques including talonavicular arthrodesis, talonaviculocuneiform arthrodesis, double arthrodesis, and triple arthrodesis have also been described (22–25). However, individual treatment for MWD was not mentioned in these reports. In these published articles, single or two compared methods were reported.

We suggest that all the adjacent joints of the navicular bone should be considered, thus, preoperative CT or MRI was essential for understanding which joint of the foot was affected and which joint should be fused to relieve the pain. CT is useful in evaluating bone destruction, the assessment of deformity, and tarsal bone arthritis. MRI is useful in detecting the disease by showing bone marrow edema, homogeneous loss of signal intensity in the dorsolateral bone marrow, and effusion in adjacent joints (7, 8). We believed that there was a higher incidence for adjacent joint arthritis in the Stage IV MWD than in the earlier stages because *pes planus* foot happened in stage IV. CT and MRI can identify adjacent joint arthritis in the stage such as calcaneal cuboid bone arthritis (Figures 1, 2). As a result, three patients in our report were revealed with calcaneal cuboid joint arthritis by CT and MRI and underwent TN plus CC fusion, and had good clinical outcomes. Otherwise, the pathological changes in the calcaneal-cuboid joint could be neglected in the treatment.

Despite regaining a stable joint and settling the tarsal joint arthritis, the goal of surgical treatment is a plantigrade, well-aligned foot with the restoration of the medial column height. We used several radiographic parameters to access the alignment of the MWD feet preoperatively and postoperatively (26). The parameters were used in a report of 36 cases (24). Despite using the parameters in these reports, we also used the intersection angle of the calcaneal and tibia bone as a parameter measured in our report, and we found that all the patients had a *varus* angle referring to the tibia. In our report, there was a statistically increase of TM lat after the surgery. There were decreased TC lat, increased CP, decreased TM ap, and decreased calcaneal *varus* angle postoperatively, but no statistical difference was shown in the parameters, and we considered that it may be due to the small number of cases. Our report showed that the alignment of the MWD feet could be improved by releasing and reducing the talus and navicular bone in fusion surgery.

## Conclusions

Our report showed that the fusion of the talus-navicular joint and the adjacent affected joint provided good clinical outcomes. CT/MRI scans are helpful to identify the adjacent joint lesion and help to decide which joint should be fused preoperatively. We recommend the application of radiographic parameters including TM lat, CPA, lateral TCA, and CAV to describe the alignment of the foot in MWD. However, there were limitations in the current study. This is a retrospective study, and a prospective study is needed to reduce the study bias. The number of cases was small due to the rareness of the disease and the follow-up duration was still not long enough to observe the subsequent periarticular degenerative changes. In addition, surgical heterogeneity may also lead to study bias in the present study.

## Data Availability Statement

The original contributions presented in the study are included in the article/supplementary material, further inquiries can be directed to the corresponding author/s.

## Ethics Statement

This study was approved the Ethics Committee of Sun Yat-sen Memorial Hospital, Sun Yat-sen University. The patients/participants provided their written informed consent to participate in this study.

## Author Contributions

WL, MM, and WS designed the study and wrote the manuscript. WL and YC collected the data and analyzed the data. GZ, MM, and TY edited the drafted manuscript. All authors approved the manuscript for submission.

## Funding

This study was supported by Sun Yat-sen Clinical Research Cultivation Program of Sun Yat-sen Memorial Hospital, Sun Yat-sen University (SYS-Q-202105), and Medical Research Foundation of Guangdong Province (A2021280).

## Conflict of Interest

The authors declare that the research was conducted in the absence of any commercial or financial relationships that could be construed as a potential conflict of interest.

## Publisher's Note

All claims expressed in this article are solely those of the authors and do not necessarily represent those of their affiliated organizations, or those of the publisher, the editors and the reviewers. Any product that may be evaluated in this article, or claim that may be made by its manufacturer, is not guaranteed or endorsed by the publisher.

## Abbreviations

AOFAS: American Orthopedic Foot and Ankle Society
AP: anteroposterior
CAV: calcaneus *varus* angle
CPA: calcaneal pitch angle
CT: computed tomography
M-T: Meary-Tomeno
MRI: magnetic resonance imaging
MWD: Müller-Weiss disease
TAC lat: lateral talocalcaneal angle
TCA ap: talocalcaneal angle
TM ap: talar–first metatarsal angle
TM lat: Tomeno-Méary angle
TN: talo-navicular
TNC: talonavicular-cuneiform
VAS: visual analog scale.
